# miR-145a-5p Promotes Myoblast Differentiation

**DOI:** 10.1155/2016/5276271

**Published:** 2016-04-28

**Authors:** Jingjing Du, Qiang Li, Linyuan Shen, Huaigang Lei, Jia Luo, Yihui Liu, Peiwen Zhang, Qiang Pu, Yi Zhang, Surong Shuai, Xuewei Li, Shunhua Zhang, Li Zhu

**Affiliations:** ^1^College of Animal Science and Technology, Sichuan Agricultural University, Chengdu 611130, China; ^2^Sichuan Province General Station of Animal Husbandry, Chengdu 611130, China; ^3^Department of Agricultural, Food and Nutritional Science, University of Alberta, Edmonton, AB, Canada T6G 2P5; ^4^Department of Animal Science, Xichang College, Xichang 61500, China

## Abstract

MicroRNAs are a class of 18–22-nucleotide noncoding RNAs that posttranscriptionally regulate gene expression and have been shown to play an important role during myoblast differentiation. In this study, we found that the expression of miR-145a-5p was gradually increased during C2C12 myoblast differentiation, and miR-145a-5p inhibitors or mimics significantly suppressed or promoted the relative expression of specific myogenesis related marker genes. Moreover, overexpression or inhibition of miR-145a-5p enhanced or repressed the expression of some special genes involved in the endogenous Wnt signaling pathway during C2C12 myoblast differentiation, including* Wnt5a*,* LRP5*,* Axin2*, and *β-catenin*. These results indicated that miR-145a-5p might be considered as a new myogenic differentiation-associated microRNA that can promote C2C12 myoblast differentiation by enhancing genes related to myoblasts differentiation.

## 1. Introduction

Skeletal muscle is the major storage tissue for postprandial glucose. Indeed, approximately 75% of insulin-dependent glucose in the plasma is stored in the skeletal muscle [1]. In addition, as a major site of metabolic activity, the skeletal muscle is a remarkably complex organ constituted by many different cell types, playing important functional roles in injury, disease, and regeneration [2, 3]. Therefore, to explore the mechanism of muscle development may contribute to a better understanding of these diseases and muscle development. Previous studies showed that some transcription factors could coordinate the expression of genes involved in muscle growth, morphogenesis, differentiation, and contractility, such as* MyoD*,* MyoG*,* myf5*,* MRF4*,* ACTA1*, and* MyHC* [4, 5]. In addition, skeletal muscle development involves several signaling pathways. For instance, the Wnt signaling pathway that involves members of the Wnt family initiates the myogenic differentiation in the epaxial and hypaxial musculature [6, 7]. MicroRNAs are a class of approximately 18−22-nucleotide, single-stranded, noncoding small RNA molecules that posttranscriptionally regulate gene expression and play important roles in cell embryogenesis, cell differentiation, carcinogenesis, tumorigenesis, and apoptosis [8, 9]. Recently, the involvement of miRNAs in the regulation of skeletal muscle differentiation has been demonstrated [10, 11], revealing that miR-145 regulates smooth muscle fate and plasticity, taking part in diverse biological processes through Wnt signaling pathway [12, 13]. However, little is known about whether miR-145a-5p plays a role in the process of myogenic differentiation. Moreover, a connection between miR-145a-5p and Wnt signaling pathway during myogenic differentiation has not been yet fully demonstrated and described.

C2C12 myoblast cells are a common model to study muscle differentiation [14–17]. In the present study, we showed that the expression level of miR-145a-5p was gradually increased during C2C12 myoblast differentiation, and reducing or increasing miR-145a-5p significantly suppressed or enhanced the relative expression levels of marker genes, when transfecting miR-145a-5p inhibitors or mimics into C2C12 cells. Additionally, the transfection of miR-145a-5p inhibitors or mimics repressed or increased the expression of genes involved in the endogenous Wnt signaling pathway during C2C12 myoblast differentiation, when compared to negative control. These data indicated that miR-145a-5p might be characterized as a new myogenic differentiation-associated microRNA and can promote C2C12 myoblast differentiation by enhancing genes related to myoblast differentiation.

## 2. Materials and Methods

### 2.1. Cell Culture

C2C12 myoblasts (Stem Cell Bank, Chinese Academy of Sciences) were maintained at 37°C and 5% CO_2_ in growth medium containing Dulbecco's modified Eagle's medium (DMEM, Gibco, Carlsbad, CA, USA) with 10% fetal bovine serum before being induced to differentiate. When cells reached 80% confluence, they were digested with 0.25% trypsin and then seeded in 12-well plates. When cell density reached 70–80%, the medium was switched to differentiation medium containing DMEM and 2% horse serum (Gibco).

### 2.2. Transfection of miR-145-5p Inhibitors, Mimics, and Negative Control

When the density of C2C12 myoblasts in 12-well plates reached 70–80%, cells were subjected to serum starvation for 4 h prior to switching the medium to differentiation medium containing DMEM and 2% horse serum. At the same time, miR-145a-5p inhibitors (30 nM; catalog number: miR20004534-1-5), mimics (30 nM; catalog number: miR10000157-1-5), and negative control (NC; catalog number: miR04101-1-2 or miR01201-1-5) (all purchased from RiboBio, Guangzhou, China) were transfected into the C2C12 myoblasts using Lipofectamine 2000 (Invitrogen, Guangzhou, China). Cell differentiation medium change and transfection were carried out every 48 h to ensure success. miR-145a-5p inhibitor was 5′-agggauuccugggaaaacuggac-3′; mimics were 5′-guccaguuuucccaggaaucccu-3′; inhibitors negative control was 5′-caguacuuuuguguaguacaaa-3′; mimics negative control was 5′-uuuguacuacacaaaaguacug-3′, 5′-caguacuuuuguguaguacaaa-3′.

### 2.3. RNA Isolation, Real-Time Polymerase Chain Reaction (PCR), and Quantitative Real-Time PCR

Total cellular RNAs (including microRNAs) were extracted using TRIzol reagent (Invitrogen, Guangzhou, China) according to the manufacturer's instruction, and the total RNA quality and concentration were estimated using denatured gel electrophoresis and a spectrophotometer (Thermo, Waltham, MA, USA). Reverse transcription of mRNA and microRNA was performed using a commercial kit (TaKaRa, China), according to the manufacturer's instructions. Quantitative real-time PCR (qRT-PCR) of mRNAs and microRNAs reactions was performed using a SYBR Premix Ex Taq kit (TaKaRa, China) on a Bio-Rad IQ*™*5 system (Bio-Rad, Hercules, CA, USA). Relative expression levels of mRNAs and microRNAs were calculated using 2^−ΔΔCt^ method. The primer sequences used for qRT-PCR are listed in Table 1. Beta-actin was used to normalize the expression levels of individual mRNAs, while microRNA expression was normalized against the expression of U6.

### 2.4. Immunocytochemical Analysis

After transfection and induced myogenic differentiation, C2C12 myoblasts cultured in 12-well plates were washed three times with phosphate-buffered saline (PBS) and fixed in 4% paraformaldehyde for 15 min. After further PBS washes (and for each step thereafter), cells were then permeabilized with 0.5% Triton X-100 prior to blocking in 2% goat serum (diluted in PBS). After blocking, cells were incubated with an anti-myosin primary antibody at 37°C for 2 h and then fluorescent secondary antibodies at 37°C for 1 h. The nuclei were stained with Hoechst (Boster, Wuhan, China) for 10 min. Images were captured using a Nikon TE2000 microscope (Nikon, Tokyo, Japan).

### 2.5. Statistical Analysis

All data are presented as means ± standard error of the mean (SEM). SPSS 22.0 software was used for statistical analysis. Comparisons were made using the one-way analysis for the least three parametric groups, and Student's *t*-test was used for two parametric groups. A value of *P* < 0.05 indicated a significant difference.

## 3. Results and Discussion

### 3.1. Expression of miR-145a-5p during the Differentiation of Mouse Myoblast

**Figure 1:**
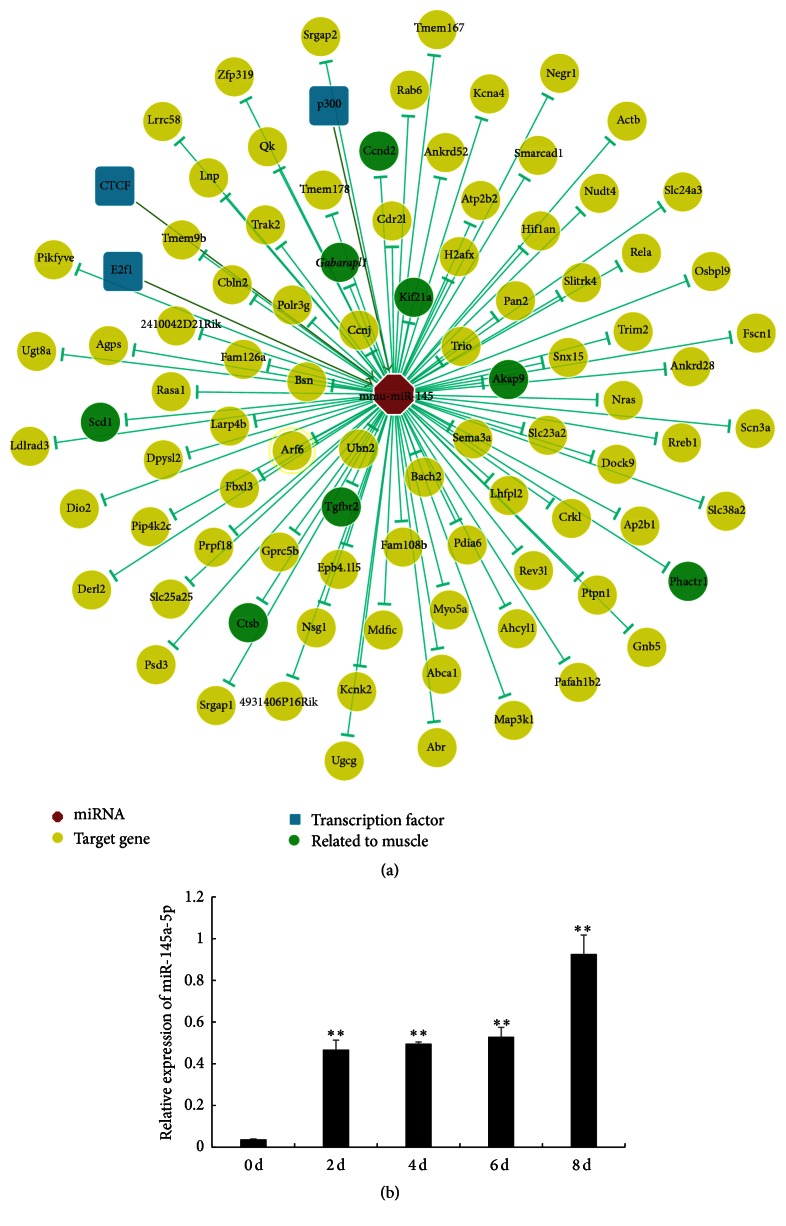
miR-145a-5p expression, targeted genes, and transcription factors during the differentiation of mouse myoblast. (a) miR-145 targeted genes and transcription factors that have been identified using experiment data from ChIPBase. (b) The expression of miR-145a-5p during C2C12 myoblast differentiation at day 0 (0 d), day 2 (2 d), day 4 (4 d), day 6 (6 d), and day 8 (8 d). Results are shown as mean ± SEM. *n* = 3 and statistical analysis was performed using one-way ANOVA; ^*∗∗*^ *P* < 0.01.

As shown in Figure 1(a), we checked some miR-145 targeted genes or transcription factors that have been identified using experiment data from ChIPBase (http://deepbase.sysu.edu.cn/chipbase/index.php). In particular, some of them are involved in muscle development, especially in myoblast differentiation (Supplementary Table S1, in Supplementary Material available online at http://dx.doi.org/10.1155/2016/5276271). To explore the potential role of miR-145a-5p in myoblast differentiation, we analyzed the temporal expression pattern of miR-145a-5p during C2C12 myoblast differentiation. As shown in Figure 1(b), as compared to zero day of the differentiation, the expression levels of miR-145a-5p in C2C12 were increased more than 25 times during differentiation (*P* < 0.01). This result showed a similar fluctuation to what was observed in miR-148a [18] and miR-486 [19]; both of them were upregulated during myoblast differentiation and played important roles in the process. Therefore, the increased expression of miR-145-5p could be also associated with miR-145a-5p important role in C2C12 cells myoblast differentiation.

### 3.2. Role of miR-145a-5p in Mouse Myoblast Differentiation

**Figure 2:**
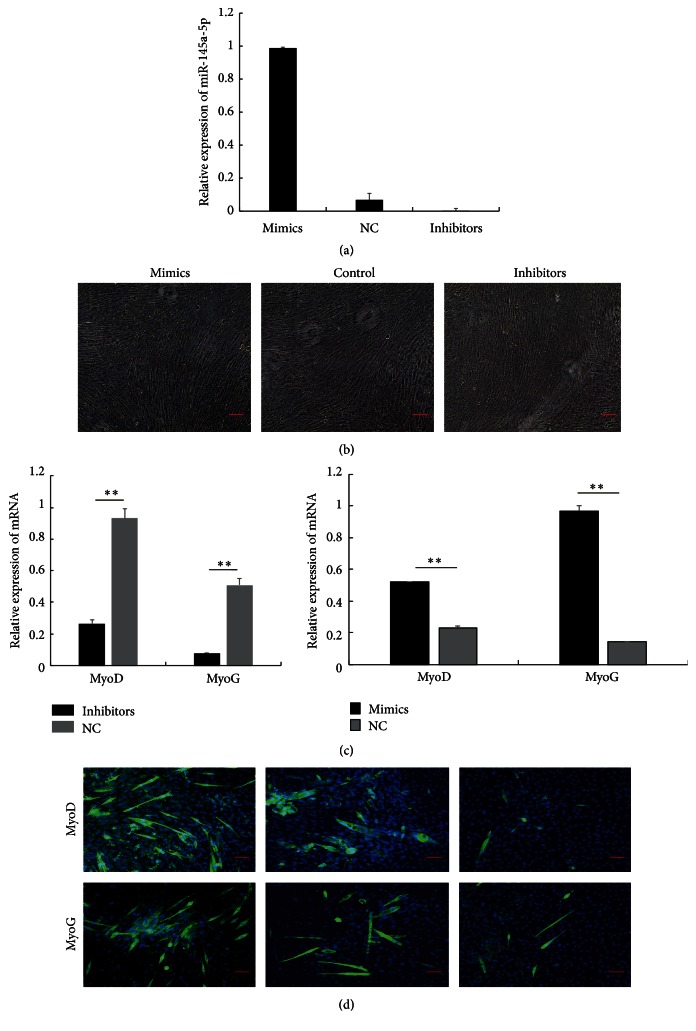
Role of miR-145a-5p in myoblast differentiation. (a) The expressions of miR-145a-5p in the negative control (NC) and in the C2C12 cells transfected with synthesized miR-145a-5p inhibitors and mimics were measured by real-time qPCR. (b) Effect of miR-145-5p on myotubes formed in C2C12 cells. Scale bar: 10 *μ*m. (c) After the C2C12 myoblasts were transfected with the synthesized miR-145a-5p inhibitors, mimics, or NC, the relative mRNA levels of* MyoD* and* MyoG* on the 8th day of differentiation were detected by real-time PCR. (d) Immunofluorescence test of* MyoD* and* MyoD* in the C2C12 myoblasts on the 8th day of differentiation. Scale bar: 100 *μ*m. The significance between the two groups at the same time point was analyzed using Student's *t*-test. Results are shown as mean ± SEM. *n* = 3; ^*∗∗*^ *P* < 0.01.

In order to discover the role of miR-145a-5p during myogenic differentiation, we firstly transfected miR-145a-5p inhibitors, mimics, or negative control (NC) into C2C12 cells during myoblasts differentiation (Figure 2(a)). As shown in Figure 2(b), miR-145a-5p successfully promoted the formation of myotube. And then we examined the expression of MyoD and MyoG on the 8th day of differentiation (Figure 2(c)). As compared with the control group, miR-145a-5p inhibitors caused a significantly progressive decrease in relative mRNA levels of* MyoD* and* MyoG*. By contrast, transfection of miR-145a-5p mimics markedly increased their expression levels compared to the negative control. Previous results showed that* MyoD* and* MyoG* are master myogenic transcriptional regulatory factors that activate a number of muscle-specific genes to drive muscle cell differentiation [20, 21]. To further investigate the role of miR-145a-5p in myogenic differentiation, we performed the immunofluorescence of* MyoD* and* MyoG* (Figure 2(d)). Similarly, myotubes were significantly decreased or increased in the cells expressing the miR-145a-5p inhibitors or mimics as compared to the control group on the 8th day after transfection.

**Figure 3:**
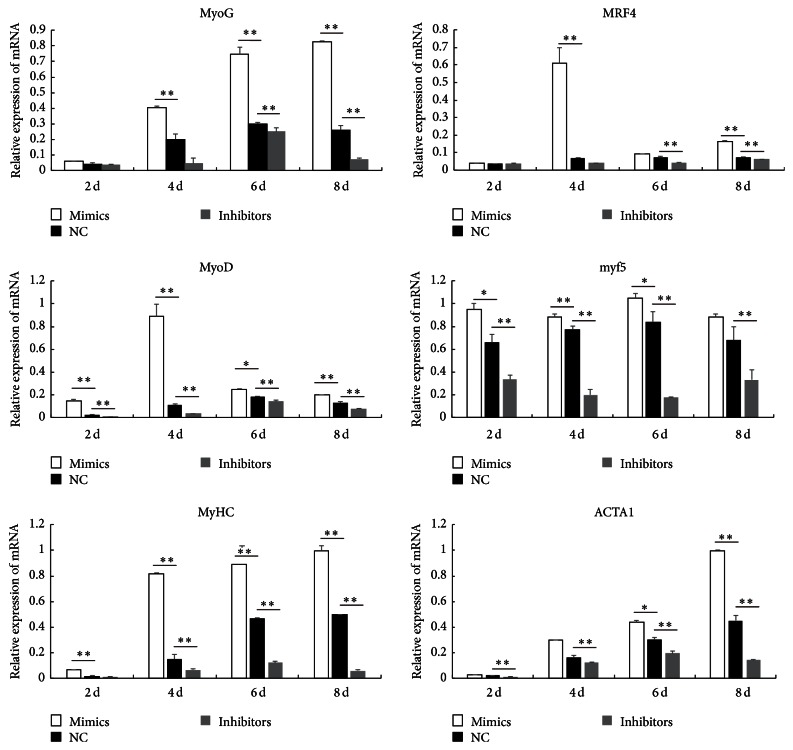
Effect of miR-145-5p on the expression of marker genes during the myoblast differentiation. After the C2C12 cells were transfected with the synthesized miR-145a-5p inhibitors, mimics, or NC, the expression of marker genes at day 2 (2 d), day 4 (4 d), day 6 (6 d), and day 8 (8 d) was measured by real-time qPCR. Results are shown as mean ± SEM. *n* = 3; ^*∗*^ *P* < 0.05; ^*∗∗*^ *P* < 0.01.

To better understand the mechanism of miR-145a-5p during myoblasts differentiation, we analyze the expression levels of more marker genes related to myoblasts differentiation. As shown in Figure 3, the expression levels of genes related to myoblasts differentiation were downregulated during myoblast differentiation, when miR-145a-5p was inhibited in C2C12 cells. However, expression levels of those maker genes were markedly increased in cells transfected with miR-145a-5p mimics, when compared to the control group.* myf5*,* MyoD*,* MyoG*,* MRF4*,* ACTA1*, and* MyHC* are key regulation factors during myoblasts differentiation [4, 22]. Previous studies showed that miR-181 indirectly upregulated the expression of* MyoD* to enhance myoblasts differentiation, while miR-139 inhibited myoblasts differentiation by repressing the expression of* MyoG* and* MyHC* [23, 24]. In the current study, almost all results supported our hypothesis that miR-145a-5p may promote the C2C12 myoblasts differentiation by enhancing some special genes related to myoblasts differentiation.

### 3.3. The Effect of miR-145a-5p on Wnt Pathway

**Figure 4:**
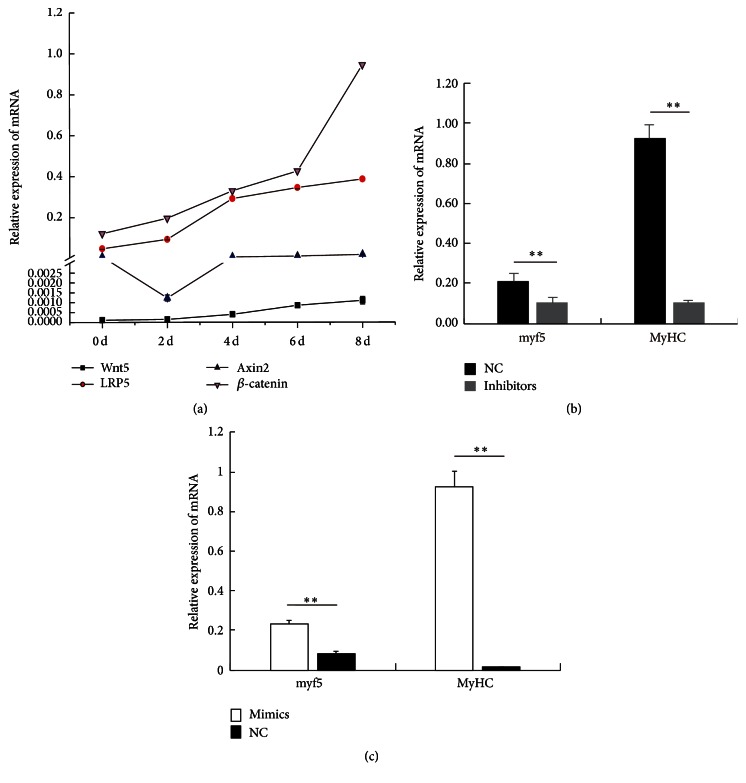
The expression of the main upstream factors of the canonical Wnt signaling pathway during myoblast differentiation. (a) Relative mRNA levels of Wnt5a, LRP5, Axin2, and *β*-catenin at day 2 (2 d), day 4 (4 d), day 6 (6 d), and day 8 (8 d) of differentiation were detected by real-time PCR. (b-c) Relative mRNA levels of myf5 and MyHC on the 8th day of differentiation, when transfecting miR-145a-5p inhibitors, mimics, or NC into C2C12 cells. Results are shown as mean ± SEM. *n* = 3. Statistical analysis was performed using Student's *t*-test; ^*∗∗*^ *P* < 0.01.

**Figure 5:**
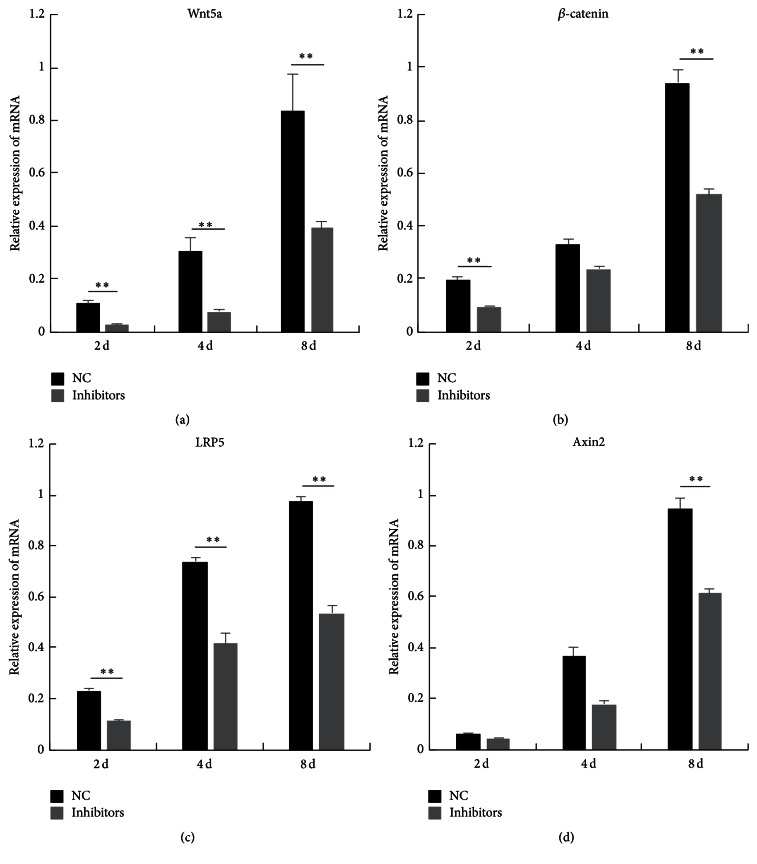
The effect of miR-145a-5p inhibitors on canonical Wnt pathway. After the C2C12 cells were transfected with the synthesized miR-145a-5p inhibitors or NC, relative mRNA levels of Wnt5a, LRP5, Axin2, and *β*-catenin at day 2 (2 d), day 4 (4 d), day 6 (6 d), and day 8 (8 d) of differentiation were detected by real-time PCR. Results are shown as mean ± SEM. *n* = 3. Statistical analysis was performed using Student's *t*-test; ^*∗∗*^ *P* < 0.01.

**Figure 6:**
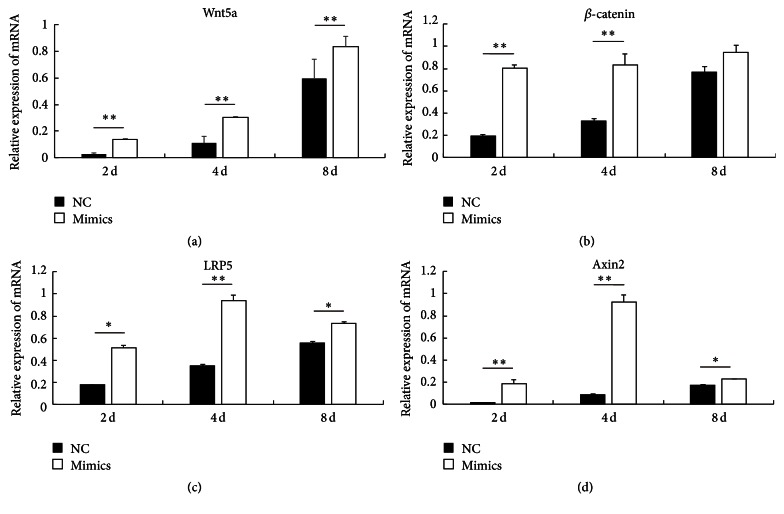
The effect of miR-145a-5p mimics on canonical Wnt pathway. After the C2C12 cells were transfected with the synthesized miR-145a-5p mimics or NC, relative mRNA levels of Wnt5a, LRP5, Axin2, and *β*-catenin at day 2 (2 d), day 4 (4 d), day 6 (6 d), and day 8 (8 d) of differentiation were detected by real-time PCR. Results are shown as mean ± SEM. *n* = 3. Statistical analysis was performed using Student's *t*-test; ^*∗*^ *P* < 0.05; ^*∗∗*^ *P* < 0.01.

Previous studies showed that miR-145 takes part in biological processes by regulating Wnt signaling in cancer [12, 13]. However, a potential connection between miR-145a-5p and Wnt signaling pathway during myogenic differentiation is not yet clearly demonstrated. As shown in Figure 4(a), we analyzed the expression of the main upstream factors of the canonical Wnt signaling pathway at the different time points before and after C2C12 cell differentiation. The mRNA levels of* Wnt5a*,* LRP5*,* Axin2*, and *β-catenin* gradually increased with the increased C2C12 differentiation. In addition, as expected, inhibiting or enhancing miR-145a-5p resulted in a significant decrease or increase in the expression levels of* Wnt5a*,* LRP5*,* Axin2*, and *β-catenin* (Figures 5 and 6). Skeletal muscle development is a complex process, involving several signaling pathways. Some previous studies indicated that Wnt signaling regulates myogenic differentiation in the development of avian wings [25], and it is involved in the switching from cell proliferation to myogenic differentiation of mouse myoblast cells [26]. In particular,* Wnt5a* can activate both* myf5* and* MyoD* [27]. In the present study, miR-145a-5p inhibitors or mimics significantly inhibited or enhanced the expression of MyHC and myf5 (Figures 4(b) and 4(c)) and the main upstream factors of the canonical Wnt signaling pathway during myoblast differentiation. This result indicated that miR-145a-5p may promote the C2C12 myoblasts differentiation and possessed a connection with Wnt signaling pathway during myogenic differentiation.

## 4. Conclusions

In the present study, we identified miR-145a-5p as a new myoblast differentiation-associated miRNA in C2C12 cells. miR-145a-5p not only possessed a positive effect on C2C12 myoblast differentiation but also promoted the expression of genes (Wnt5a, LRP5, Axin2, and *β*-catenin) involved in the endogenous Wnt signaling during C2C12 myoblast differentiation. These results suggested that miR-145a-5p may promote the expression of specific myoblast differentiation markers and myotube differentiation. From a clinical point of view, miR-145a-5p could also be considered as a novel disease marker for the diagnosis of muscle wasting or for monitoring its progression, leading to useful therapies for this type of disease.

## Supplementary Material

As shown in the supplementary material, you will find some gene involved in muscle development, in particular, myoblasts differentiation, which have been identified with experiments.

## Figures and Tables

**Table 1:** The primer sequences used for qRT-PCR. F: forward; R: reverse.

Gene	Primer sequence (5′→3′)	TM °C
MyoD	F: AGACTTCTATGATGACCCGTGTT	58
	R: TCAGCGTTGGTGGTCTTGC	
MyoG	F: GCCCAGTGAATGCAACTCCCACA	58
	R: CAGCCGCGAGCAAATGATCTCCT	
myf5	F: GAGCTGCTGAGGGAACAGGTGGAGA	58
	R: GTTCTTTCGGGACCAGACAGGGCTG	
MRF4	F: ATTCTTGAGGGTGCGGATTTCCTG	58
	R: AAGACTGCTGGAGGCTGAGGCATC	
ACTA1	F: CCTTTATCGGTATGGAGTCTGCG	58
	R: CCTGACATGACGTTGTTGGCA	
Wnt5a	F: TACATTGGAGAAGGTGCGAAGA	58
	R: TCTCTCGGCTGCCTATTTGC	
LRP5	F: CAGCACCACAAGCCACCAA	58
	R: TCCCTTCATACGAGGACACAGC	
Axin2	F: AGTCCCTCCTTACCGCATGG	58
	R: AGCAGGTTCCACAGGCGTCA	
*β*-catenin	F: TGCTGGTGACAGGGAAGACATC	58
	R: GATGGTGGGTGCAGGAGTTTAA	
miR-145a-5p	GUCCAGUUUUCCCAGGAAUCCCU	61
Beta-actin	F: CAGCCTTCCTTCTTGGGTAT	58
	R: TGGCATAGAGGTCTTTACGG	
U6	F: CTCGCTTCGGCAGCACA	58
	R: AACGCTTCACGAATTTGCGT	
