# Supplementary material for: miR-145a-5p Promotes Myoblast Differentiation
Source: Biomed Res Int. 2016 Apr 28;2016:5276271. doi: 10.1155/2016/5276271 (PMC4864570; doi:10.1155/2016/5276271)
Supplement: Supplementary file 1 — As shown in the supplementary material, you will find some gene involved in muscle development, in particular, myoblasts differentiation, which have been identified with experiments. [file 5276271.f1.pdf]

Gene	Full name	Function	References
Gabarap1	GABA receptor-associated protein 1	Involve in contractile dysfunction of muscle and muscle wasting	(1, 2)
Kif21a	Kinesin family member 21A	Relate to muscle fibrosis	(3)
Akap9	A kinase-anchoring protein 9	Involve in myoblast differentiation and airway smooth muscle secretory function	(4)
Ccnd2	Cyclin D2	Enhance myogenic differentiation and induce regeneration	(5)
Ctsb	Cathepsin B	Relate to muscle proteolytic system	(6)
Phactr1	The protein phosphatase and actin regulator 1	Relate to G-Actin	(7)
Scd1	Stearoyl-CoA desaturase 1	Regulate glucose metabolism of muscle	(8, 9)
Tgfbr2	The type II transforming growth factor β receptor	Regulate smoth muscle differentiation and skeletal development	(10, 11)

Table S1: Genes related to the muscle development.

References:

[1]. Gaugler M, Brown A, Merrell E, DiSanto-Rose M, Rathmacher JA, Reynolds TH. “PKB signaling and atrogene expression in skeletal muscle of aged mice,” *Journal of Applied Physiology*, vol. 111, n. 1, pp. 192-199, 2011.

[2]. Lecker SH, JAGOE RT, GILBERT A, GOMES M, BARACOS V, BAILEY J, et al. “Multiple types of skeletal muscle atrophy involve a common program of changes in gene expression,” *The FASEB Journal*, vol. 18, n. 1, pp. 39-51, 2004.

[3]. Yamada K, Andrews C, Chan W-M, McKeown CA, Magli A, de Berardinis T, et al. “Heterozygous mutations of the kinesin KIF21A in congenital fibrosis of the extraocular muscles type 1 (CFEOM1),” *Nature genetics*, vol. 35, n. 4, pp. 318-321, 2003.

[4]. Karolczak J, Sobczak M, Skowronek K, Rędowicz MJ. “A Kinase Anchoring Protein 9 Is a Novel Myosin VI Binding Partner That Links Myosin VI with the PKA Pathway in Myogenic Cells,” *BioMed research international*, vol. 2015, Article ID 816019, 2015.

[5]. Khanjyan MV, Yang J, Kayali R, Caldwell T, Bertoni C. "A high-content, high-throughput siRNA screen identifies cyclin D2 as a potent regulator of muscle progenitor cell fusion and a target to enhance muscle regeneration," *Human molecular genetics*. vol. 22, n. 16, pp. 3283-3295, 2013.

[6]. Nishimura M, Mikura M, Hirasaka K, Okumura Y, Nikawa T, Kawano Y, et al. "Effects of dimethyl sulphoxide and dexamethasone on mRNA expression of myogenesis-and muscle proteolytic system-related genes in mouse myoblastic C2C12 cells," *Journal of biochemistry*, vol. 144, n. 6, pp. 717-724, 2008.

[7]. Mouilleron S, Wiezlak M, O'Reilly N, Treisman R, McDonald NQ. "Structures of the Phactr1 RPEL domain and RPEL motif complexes with G-actin reveal the molecular basis for actin binding cooperativity," *Structure*, vol. 20, n. 11, pp. 1960-1970, 2012.

[8]. Rogowski MP, Flowers MT, Stamatikos AD, Ntambi JM, Paton CM. "SCD1 activity in muscle increases triglyceride PUFA content, exercise capacity, and PPARδ expression in mice," *Journal of lipid research*, vol. 54, n. 10, pp. 2636-2346, 2013.

[9]. Parra P, Serra F, Palou A. "Transcriptional analysis reveals a high impact of conjugated linoleic acid on stearoyl-Coenzyme A desaturase 1 mRNA expression in mice gastrocnemius muscle," *Genes & nutrition*, vol. 7, n. 4, pp. 537-548, 2012.

[10]. Frutkin AD, Shi H, Otsuka G, Levéen P, Karlsson S, Dichek DA. "A critical developmental role for tgfbr2 in myogenic cell lineages is revealed in mice expressing SM22-Cre, not SMMHC-Cre," *Journal of molecular and cellular cardiology,* vol. 41, n. 4, pp. 724-731, 2006.

[11]. Choudhary B, Ito Y, Makita T, Sasaki T, Chai Y, Sucov HM. "Cardiovascular malformations with normal smooth muscle differentiation in neural crest-specific type II TGFβ receptor (Tgfbr2) mutant mice," *Developmental biology*, vol. 289, n. 2, pp. 420-429, 2006.
